# “Networks” is Different

**DOI:** 10.3389/fgene.2012.00183

**Published:** 2012-09-19

**Authors:** Roger Guimerà

**Affiliations:** ^1^Institució Catalana de Recerca i Estudis Avançats (ICREA)Barcelona, Spain; ^2^Departament d'Enginyeria Química, Universitat Rovira i VirgiliTarragona, Spain

A cell is more than a collection of molecules, as much as a brain is more than a collection of neurons and an ecosystem is more than a collection of species. This argument, which was first formalized by Anderson (1972) in his seminal “More is different,” is now evident to most of us. At the same time, however, we still aim to identify a single gene responsible for such and such type of cancer, and we hope (or hoped) that the mere identification of all human genes would immediately open the door to revolutionary medical advances.

One of the reasons why such grand expectations are misplaced is that individual components in complex systems (for example, proteins in a cell or neurons in the brain) interact with each other through intricate networks. The promise of Network Science is to help identify how networks determine the emergent behavior of systems (and, particularly, biological systems); and to provide tools to extract useful information from those networks (for example, about the evolutionary constraints that have shaped the system).

**Figure 1:**
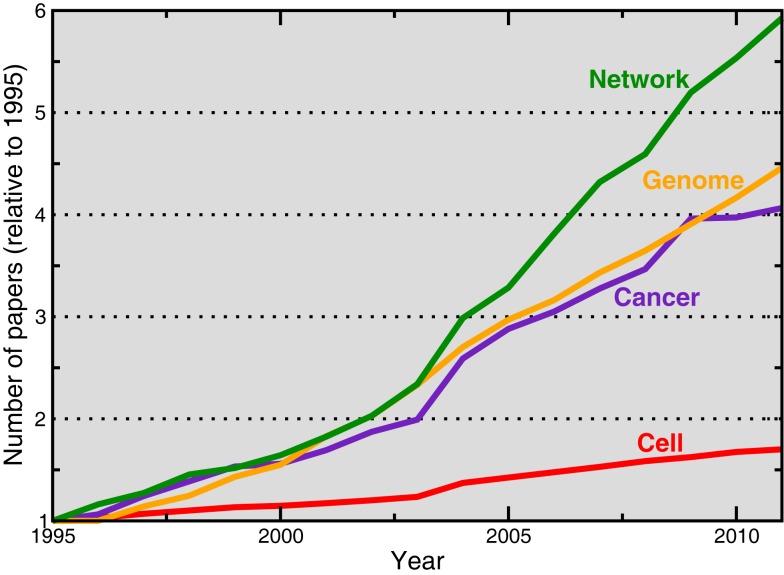
**Evolution of the number of articles in the life sciences about different topics, including networks**. The data was obtained by querying the “Topic” field in the ISI Web of Science with the corresponding terms (for example, “network or networks” or “cancer”), and including publications in areas such as Biochemistry and Molecular Biology, Cell Biology, Genetics and Heredity, Neurosciences, or Pharmacology.

With such an ambitious program, Network Science is a fast growing discipline with an increasing number of applications in the life sciences – from molecular and cellular biology, to medicine and pharmacology, the number of papers using network methods is growing fast (Figure 1). Maybe surprisingly, up to now there was not a textbook that covered the fundamentals of the theoretical and computational tools that network scientists use on a daily basis. The book “Networks: An introduction,” by Newman (2011), does a beautiful job at filling the gap between more or less advanced popular science books about networks, and highly specialized volumes on specific network topics (often in the form of collections of disconnected articles). As Network Science graduate-level courses proliferate around the world, this book is very likely to become a classic textbook, and deservedly so. The book is well written and clarity is put ahead of everything else.

The book is organized in five parts. The first part provides a broad overview of areas where network science has been successfully used, including the social sciences, information technology, and biology. The second and third parts are the core of the book, and give a thorough account of the theoretical and algorithmic tools that network scientists have developed, mostly during the last decade and a half, to characterize networks. Part III, devoted specifically to the description of computer algorithms, is particularly useful considering that this is an aspect that has often been disregarded as “technical” and uninteresting (and that many network scientists have had to learn the hard way, after discovering that a calculation that was taking months to complete could actually be carried out in seconds).

The fourth part of the book is devoted to network models. The models covered here are mostly abstract and intended to explain general network properties, as opposed to particular features of specific systems. Although abstract, these models have been and continue to be instrumental in shaping our understanding of network structure, function, and evolution. The last part of the book is devoted to processes “on networks,” that is, to processes that either alter the structure of networks (for example, processes involving the removal of connections or nodes) or that are critically affected by network structure (such as epidemic spreading on social networks).

A few warnings specifically directed to biologists are in order. First and foremost, this book is *not* about network biology specifically, and Mark Newman's expertise is more on social and technological networks – and, of course, on network methods and theory. Therefore, most biologists interested in networks may want to skip the chapters devoted to biological networks (which they may find overly simplifying or even confusing) and read, instead, some of the excellent books on specific biological networks that are available (for example, Alon’s, 2006 book on systems biology with an emphasis on regulation, Palsson’s, 2006, 2011 books on network biology with an emphasis on metabolism, or Sporn’s, 2010 book on brain networks). Second, some chapters are relatively heavy in terms of mathematics – although there is very little in the book that requires math beyond undergraduate-level algebra and calculus, some may find it demanding. These are not a limitations of the book itself, but a consequence of the fact that the intended audience of the book are not (only) biologists. In any case, one may argue that any scientist wishing to use network methods first hand should be able to, at least, follow most of the derivations.

In 2013, the new journal Network Science will come to life – according to the publishers, it is “a new journal for a new discipline.” Mark Newman’s “Networks: An introduction” is bound to be “a new textbook for a new discipline” – as well as a reference for those wishing to become conversational on networks and for those already working on networks who need a quick consultation from time to time.
